# A comprehensive approach for microbiota and health monitoring in mouse colonies using metagenomic shotgun sequencing

**DOI:** 10.1186/s42523-021-00113-4

**Published:** 2021-07-29

**Authors:** Ferdinando Scavizzi, Cristian Bassi, Laura Lupini, Paola Guerriero, Marcello Raspa, Silvia Sabbioni

**Affiliations:** 1 National Research Council (IBBC), CNR-Campus International Development, (EMMA-INFRAFRONTIER- IMPC), Monterotondo Scalo, Italy; 2 Department of Morphology, Surgery and Experimental Medicine, University of Ferrara, 44121 Ferrara, Italy; 3 Laboratorio Per Le Tecnologie Delle Terapie Avanzate (LTTA), University of Ferrara, 44121 Ferrara, Italy; 4 Department of Life Science and Biotechnology, University of Ferrara, Via Luigi Borsari 46, 44121 Ferrara, Italy

**Keywords:** Microbioma, NGS shotgun sequencing, Metagenomics, Gut microbiota, Laboratory mice, Health surveillance, Mouse colonies

## Abstract

**Background:**

Health surveillance of murine colonies employed for scientific purposes aim at detecting unwanted infection that can affect the well-being of animals and personnel, and potentially undermine scientific results. In this study, we investigated the use of a next-generation sequencing (NGS) metagenomic approach for monitoring the microbiota composition and uncovering the possible presence of pathogens in mice housed in specific pathogen-free (SPF) or conventional (non-SPF) facilities.

**Results:**

Analysis of metagenomic NGS assay through public and free algorithms and databases allowed to precisely assess the composition of mouse gut microbiome and quantify the contribution of the different microorganisms at the species level. Sequence analysis allowed the uncovering of pathogens or the presence of imbalances in the microbiota composition. In several cases, fecal pellets taken from conventional facilities were found to carry gene sequences from bacterial pathogens (*Helicobacter hepaticus, Helicobacter typhlonius, Chlamydia muridarum, Streptococcus pyogenes, Rodentibacter pneumotropicus, Citrobacter rodentium, Staphylococcus aureus*), intestinal protozoa (*Entamoeba muris*, *Tritrichomonas muris, Spironucleus muris*) nematoda (*Aspiculuris tetraptera, Syphacia obvelata*), eukaryotic parasites (*Myocoptes musculinus*) and RNA virus (*Norwalk virus*). Thus, the use of NGS metagenomics can reduce the number of tests required for the detection of pathogens and avoid the use of sentinel mice.

**Conclusions:**

In summary, in comparison with standard approaches, which require multiple types of test, NGS assay can detect bacteria, fungi, DNA and RNA viruses, and eukaryotic parasites from fecal pellets in a single test. Considering the need to protect animal well-being and to improve the success and reproducibility of preclinical studies, this work provides the proof-of-concept that the use of NGS metagenomics for health monitoring of laboratory mice is a feasible and dependable approach, that is able to broaden the current concept of health monitoring of laboratory mice from “pathogen surveillance” to a more inclusive “microbiota surveillance”.

**Supplementary Information:**

The online version contains supplementary material available at 10.1186/s42523-021-00113-4.

## Introduction

Health surveillance of murine colonies used for scientific purposes is based on pathogen surveillance to detect viral, bacterial, and parasitic infections. Health monitoring programs aim at detecting unwanted infections, which can affect animals and personnel welfare, and can also undermine scientific experimental results [1–4]. Traditionally, health monitoring is performed by testing sentinel animals that periodically receive dirty bedding from the other cages and therefore, represent the microbiological health status of the whole colony. Diagnosis is based on bacterial cultivation, serology, and molecular tests for the detection of viruses or uncultivable microorganisms. Microbiological, microscopic, molecular, and serological analyses are performed to assess the health status of the sentinel and the presence of pathogens, assuming that the microbiological status of the sentinel mirrors that of the entire colony.

There are limitations to this approach. On the one hand, it is assumed that all pathogens eventually present in the colony are transferred efficiently to the bedding and that this results in sentinel infection. However, the prevalent use of individually ventilated cages systems challenged this approach, as transmission of infectious agents through dirty bedding has been shown to be variable and generally insufficient [5]. Thus, employment of bedding sentinels in health monitoring programs cannot be totally justified on the basis of infectious agent transfer efficiency. Moreover, ethical reasons and enforced regulations, at least in the European Union, require that animals not be used unless absolutely necessary, and the use of mouse sentinels appears non-compliant with the reduction arm of the 3R (Replacement, Reduction, Refinement) guidelines [6].

Molecular detection (using PCR or real-time PCR) of mouse pathogens directly on colony animals is now recognized as the preferable way to proceed, having higher sensitivity than the other methods [7, 8]. However, multiple tests are necessary to identify the different pathogens, and some of them even require necropsy. Moreover, the molecular approaches currently used do not provide information on the composition of the intestinal microbiota of colony animals, which is an essential factor for the correct development of the host organism [9–24].

On the other hand, based on studies on human infectious diseases [25], high throughput metagenomic sequencing has emerged as an attractive approach for pathogen detection in clinical samples. Metagenomic next generation sequencing (mNGS) provides sequencing of all the nucleic acids present in the samples, both of the host and of microbial origin, including viruses, fungi, and parasites; thus, it is not limited to bacterial sequences detection only, as it is the case for the targeted sequencing method of the 16S rRNA gene. With respect to the single-strain PCR testing, it allows untargeted microbial identification and a comprehensive description of the sample microbiota. Moreover, it allows the discovery of new organisms [26], enables species and strain identification [27], and provides a quantitative assessment of the relative abundance of each microbial species in the investigated samples [28]. Based on this previous knowledge, we propose here the use of mNGS for health surveillance of murine colonies employed for scientific purposes, to enhance the diagnostic ability of pathogen detection, replacing a variety of targeted tests and allowing the identification of all the microorganisms composing the sample microbiota. Moreover, costs associated with this technology are becoming more affordable and are now comparable, if not advantageous, compared with the costs associated with multiple single-strain PCR testing.

The gut microbiota (GM) is transmitted to litters at birth and is then shaped by milk-derived oligosaccharides to reach maturity after weaning. It is also influenced by cage mate interactions, leading to a gradual homogenization of the gut microbiota between co-housed mice [29].

The role of GM has now been established in different pathologies in humans and in mouse models, such as obesity [12, 30–34], autoimmune and inflammatory diseases, [10, 35, 36], carcinogenesis [37–40], atherosclerosis [41], impairment of cardiac repair after myocardial infarction [11], and in the modulation of host response to therapies, e.g. anticancer treatments [42, 43]. It has been suggested that microbiota differences among facilities may be responsible for phenotype changes in genetically defined disease models and may also have an impact on the transferability of results from preclinical to clinical studies [44–48].

Thus, analysis and monitoring of the microbiota of colonies employed in scientific research is required in order to address the novel needs of breeders and researchers [49].

In this study, we analyzed the fecal samples of animals taken from Specific Pathogen Free (SPF) or from conventional (non-SPF) housing facilities. The direct fecal sampling from animals can help to overcome several limitations of the current health surveillance strategies of murine colonies used for scientific purposes. We propose mNGS as the most effective approach for monitoring microbiota composition as well as mouse pathogens, with specific attention to those reported in the Federation of European Laboratory Animal Science Associations (FELASA) list. This approach has the advantage of being continuously updated as it detects any possible form of life whose genomic sequence is present in public and continuously updated databases.

## Results

Mice employed in the study were from SPF (n = 10) and non-SPF (n = 27) housing facilities. Twenty-one mice (n = 10 SPF and n = 11 non SPF) were sentinel animals included in the institutional health program, routinely monitored to assess the health and microbiological status of the colony. Each animal provided test and control samples; test sample consisted of fecal DNA analyzed by mNGS, while control samples consisted of different tissues (fur; caecal content; blood; fecal DNA; intestinal content) analyzed for specific pathogens using different methods (microscopic observation, ELISA, PCR, culture techniques, respectively), as described in Materials and Methods. Additional mice employed in the study (n = 16, non-SPF), belonging to multiple breeding colonies, provided both test (fecal DNA analysed by mNGS) and control samples (fecal DNA analysed by PCR) (see Additional file 1). Moreover, to provide a negative control of sampling, extraction, library preparation and sequencing, a pulverized sample of chow and bedding taken from a microisolator cage without animals and placed in the IVC rack for 4 weeks, was subjected to the same DNA extraction procedure performed for the fecal samples and used for library preparation and sequencing.

We performed mNGS shotgun sequencing using nucleic acids (DNA and RNA) isolated from fecal pellet samples taken from 37 mice, as indicated above. To allow identification of RNA viruses, nucleic acids were retro-transcribed to convert RNA into cDNA before library preparation and sequencing, as described in Materials and Methods. Sequence raw data was analyzed using a pipeline shown in Additional file 2 and detailed in Materials and Methods. High-quality filtered sequence data exhibited an average of 6.7 × 10^6^ reads per sample (range: 2,071,086–15,825,000 reads). All reads less than 100 nucleotides were filtered out and only reads with a quality higher than Q30 were included. The filtered reads were used to perform taxonomy calling, from phylum to genus and species, using Kraken 2 [50], Bracken [51], on the basis of a reference database consisting of all the complete and draft genome sequences of archaea, bacteria, fungi, protozoa, virus and invertebrate endo- and ecto-parasites of mice (*Acantocephala*, *Annelida*, Helminths and *Nematoda*) present in GenBank Release 232 (Additional file 3). Sequence reads aligned to host (*mus musculus* genome version mm10) were on average 10% of the total. Of the remaining reads, about the 8% reads aligned to microbial genomes with an average 5,40,000 reads per sample, ranging from a minimum of 45,000 to a maximum of 1,200,000 reads. The negative control sequences showed species present in the control only, or abundant in the control and scarce in the samples or vice versa (Additional file 4). The negative control consists of pulverized sample of chow and bedding taken from a microisolator cage without animals. As expected, several specific taxa of the negative control are plant epiphytic bacteria (i.e. *Erwinia gerundensis, Pantoea vagan*s), plant endophytes fungi (*Fusarium oxysporum*) or plant pathogens, both bacteria (*Pectobacterium carotovorum, Pseudomonas syringae*) fungi (*Fusarium pseudograminearum, Ustilago maydis*) and viruses (*Brome mosaic virus, Wheat dwarf virus*). The negative control contains also several species of *Staphylococcus*, in accordance with reports indicating the presence of genus *Staphylococcus* in general and *S. epidermidis* in particular, as normal constituents of plant microbiome [52].

**Fig. 1:**
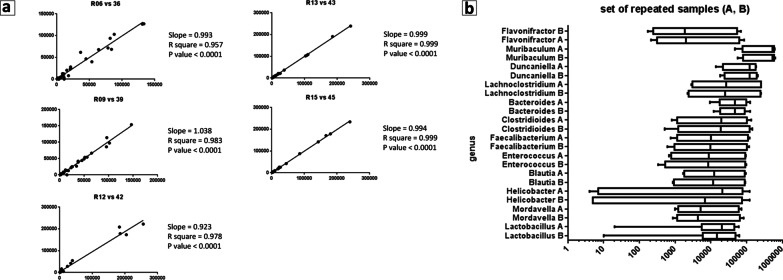
Correlation analysis for each pair of 5 re-sequenced samples. **a** Each dot represents a species, plotted from normalized counts for sample a (X-axis) and for sample b (Y-axis) of each pair. Slope, Pearson correlation coefficient (r), and p-value are shown for each pair of samples. The values of those indexes indicate that each re-sequenced pair of samples shows significant correlation. **b** Top 12 genera in the mouse gut microbiota of five samples sequenced at time A and of the same samples sequenced at time B. Genera (listed in Y-axis) are depicted with boxes including 5–95% of data, median values (dark lines in the boxes). Plots are based on normalized counts (X-axis). Outcome of the analysis in the two set of samples is almost identical

The robustness of mNGS shotgun sequencing was established by resequencing 5 samples. The repeated samples were prepared and sequenced at different times and by different operators. Data were analyzed using the same pipeline. The sequencing data (reads mapped to microbial genomes) from the two duplicates were compared. A correlation analysis for each pair of re-sequenced samples (Fig. 1a, b) revealed a Pearson correlation coefficient, r, ranging from 0.957 to 0.999, a result that indicated reproducibility and robustness of the analyses.

### Gut microbiome composition in mice from SPF and non-SPF facilities

**Fig. 2:**
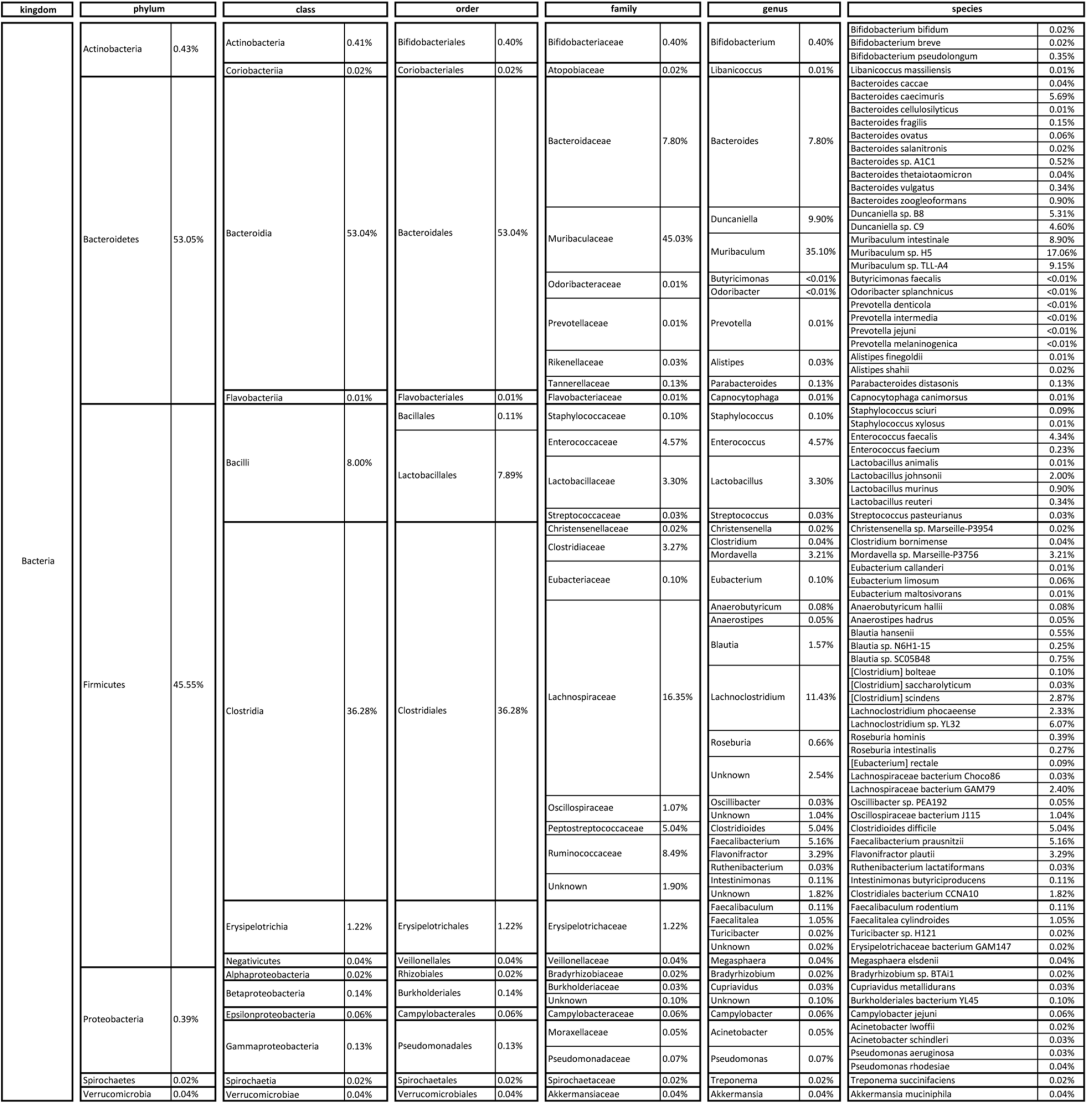
Bacteria composition of SPF samples. Taxonomy of gut bacterial microbiota of SPF samples reveals that 82 species represent more than 99% of the intestinal microbiota, belonging to 31 families, the most abundant being *Muribaculaceae* (45.03%), *Lachnospiraceae* (16.35%), *Ruminococcaceae* (8.49%), and *Bacteroidaceae* (7.8%). Bacterial composition at phylum, class, order, and genus level is also shown

With regard to SPF housed mice, sequence analyses identified over 200 bacterial species of which, 82 represented more than 99% of the intestinal microbiota species. They showed an abundance higher than > 0.01% and belonged to 31 families, within the phyla of *Bacteroidetes* (53.0%), *Firmicutes* (45.6%), *Actinobacteria*, (0.4%), *Proteobacteria* (0.4%), *Verrucomicrobia* (0.04%) and *Spirochaetes* (0.02%) (Fig. 2).

**Fig. 3:**
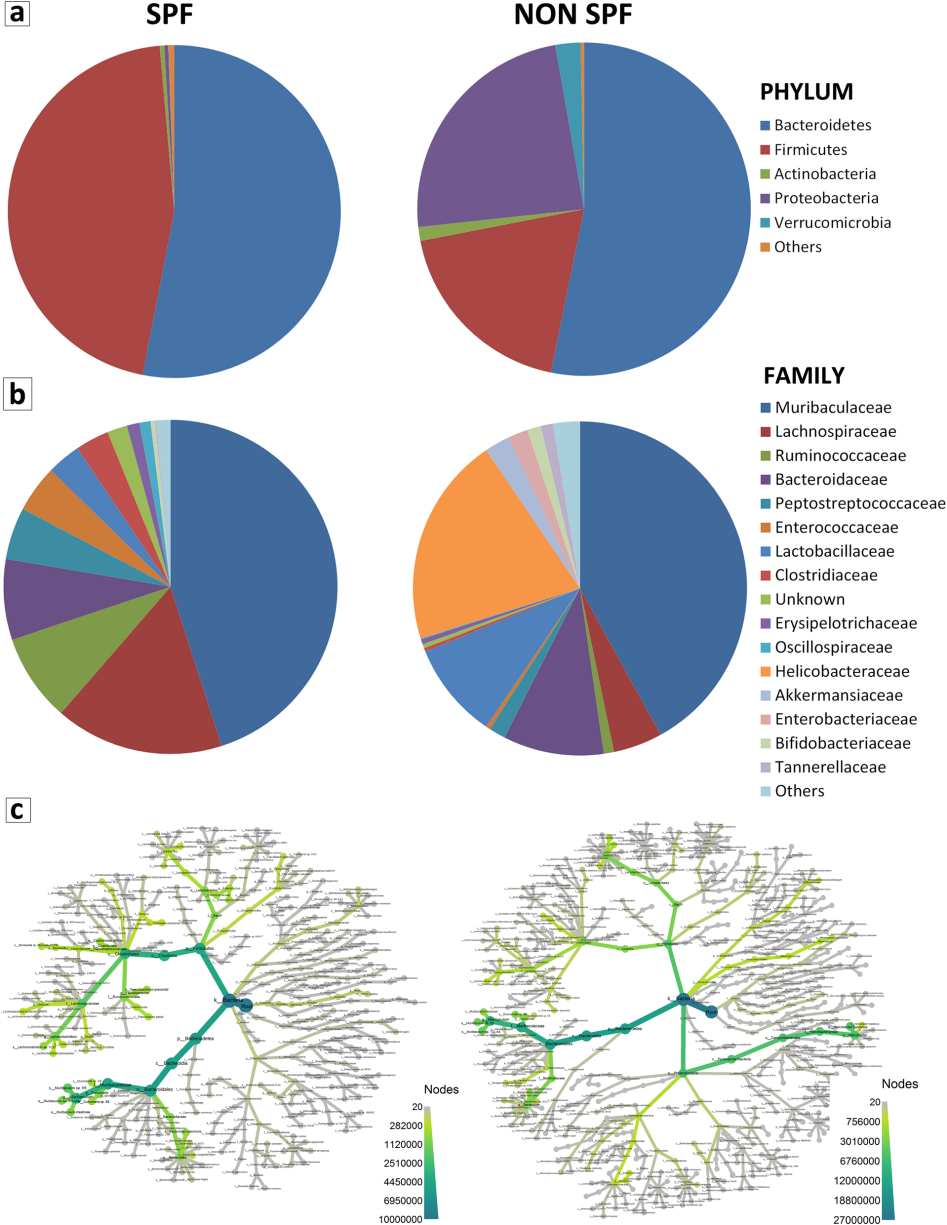
Comparison of mouse gut microbiome in SPF and non-SPF facilities. **a** Phylum level**.** Graph represents phyla (average greater than 100 ppm in at least one of the two groups of samples) in SPF and non-SPF samples: there are no differences relating to the abundance of *Bacteroidetes* (53% in both SPF and non-SPF mice), while *Firmicutes* represent 45% and 19% in SPF and non-SPF mice, respectively. *Proteobacteria* represent 0.4% of phyla in the SPF mice, while they constitute the most abundant phylum (24%) in the non-SPF mice. *Verrucomicrobia* and *Actinobacteria* are significantly more abundant in the non-SPF mice (2% and 1%, respectively) than in the SPF mice (0.04% and 0.4%, respectively). The less abundant Phyla represent 0.5% (in SPF mice) and 0.3% (in non-SPF mice) of the total. **b** Family level. Graph represents families, with average above 100 ppm in both groups of samples, except for *Helicobacteraceae* which are absent in the SPF mice, but are the second largest family (20%) in the non-SPF mice. *Muribaculaceae* is confirmed as the most abundant family in both groups (46% in the SPF and 42% in the non-SPF). *Bacteroidaceae* represents 8% and 9% and *Lactobacillaceae* 3% and 9% of families in the SPF and non-SPF mice, respectively. *Lachnospiraceae* family is reduced in the non-SPF (5%) compared to SPF (16%) mice. **c** Phylogenic tree of microbiome species**.** Images represent bacterial composition of gut microbiome in the non-SPF and SPF samples. Branches represent taxonomic classification and nodes represent transition to the subsequent taxonomy level, from super kingdom to species level. Thickness and color of branches represent abundance difference. With respect to the SPF samples, in the non-SPF ones there is a noticeable increase of *Proteobacteria* and to a lesser extent of *Verrucomicrobia,* and a decrease of *Firmicutes*. A greater number of species is also appreciable in the non-SPF samples compared to the SPF ones

Compared to SPF, the analysis of non-SPF mice revealed that the microbiome composition was similar in terms of phyla and families, with some quantitative significant differences for *Bacteroidetes, Firmicutes, Proteobacteria* and *Verrucomicrobia* among phyla and for *Bacteroidaceae, Enterococcaceae, Lactobacillaceae, Erysipelotrichaceae, Helicobacteraceae, Akkermansiaceae, Enterobacteriaceae, Bifidobacteriaceae* and *Tannerellaceae* among families (Fig. 3a, b and Additional file 7). A notable difference was the presence of bacteria belonging to the family *Helicobacteraceae* (20.3%), which was absent in the SPF mice, as indicated also by the comparison between the phylogenic trees of the two groups of samples (Fig. 3c).

Interestingly, in both cases, a relatively small number of species constituted the largest part of microorganisms. Nineteen species with an average abundance higher than 1%, which we called "SPF-core species", comprised 91.4% of bacteria present in the gut of the SPF mice (Table 1); twenty-four species with an average abundance higher than 1%, which we called "Conventional-core species" constituted 90.3% of all microorganisms present in non-SPF mice (Table 2).

**Table 1:** Main species constituting the microbiota in SPF animals

Phylum	Class	Order	Family	Genus	Species	Species ID	
Bacteroidetes (50.7%)	Bacteroidia (50.7%)	Bacteroidales (50.7%)	Bacteroidaceae (5.7%)	Bacteroides (5.7%)	*Bacteroides caecimuris*	1796613	"SPF -core species" (91.4%)
			Muribaculaceae (45%)	Duncaniella (9.9%)	*Duncaniella* sp. B8	2576606	
					*Duncaniella *sp. C9	2530392	
				Muribaculum (35.1%)	*Muribaculum intestinale*	1796646	
					*Muribaculum* sp. H5	2530393	
					*Muribaculum* sp. TLL-A4	2530390	
Firmicutes (16.6%)	Bacilli (6.3%)	Lactobacillales (6.3%)	Enterococcaceae (4.3%)	Enterococcus (4.3%)	*Enterococcus faecalis*	1351	
			Lactobacillaceae (2%)	Lactobacillus (2%)	*Lactobacillus johnsonii*	33959	
	Clostridia (33.3%)	Clostridiales (33.3%)	Clostridiaceae (3.2%)	Mordavella (3.2%)	*Mordavella* sp. Marseille-P3756	2086584	
			Lachnospiraceae (13.7%)	Lachnoclostridium (11.3%)	*[Clostridium] scindens*	29347	
					*Lachnoclostridium phocaeense*	1871021	
					*Lachnoclostridium* sp. YL32	1834196	
				Unknown (2.4%)	*Lachnospiraceae* bacterium GAM79	2109691	
			Oscillospiraceae (1%)	Unknown (1%)	*Oscillospiraceae* bacterium J115	2093857	
			Peptostreptococcaceae (5%)	Clostridioides (5%)	*Clostridioides difficile*	1496	
			Ruminococcaceae (8.6%)	Faecalibacterium (5.2%)	*Faecalibacterium prausnitzii*	853	
				Flavonifractor (3.3%)	*Flavonifractor plautii*	292800	
			Unknown (1.8%)	Unknown (1.8%)	*Clostridiales* bacterium CCNA10	2109688	
	Erysipelotrichia (1.1%)	Erysipelotrichales (1.1%)	Erysipelotrichaceae (1.1%)	Faecalitalea (1.1%)	*Faecalitalea cylindroides*	39483	

List of the nineteen species with an average abundance higher than 1% ("SPF-core species"), comprising 91.4% of bacteria present in the gut of the SPF mice. Taxonomy from phylum to specie level is shown. The different lines represent the species included in the list. For each species there are columns that describe the taxonomy (phylum, class, order, family, genus, species, Species ID)

**Table 2:** Main species constituting the microbiota in non-SPF animals

Phylum	Class	Order	Family	Genus	Species	Species ID	
Bacteroidetes (49.6%)	Bacteroidia (49.6%)	Bacteroidales (49.6%)	Bacteroidaceae (7.7%)	Bacteroides (7.7%)	*Bacteroides caecimuris*	1796613	"Non-SPF—core species" (90.3%)
			Muribaculaceae (41.9%	Duncaniella (8.7%)	*Duncaniella* sp. B8	2576606	
					*Duncaniella* sp. C9	2530392	
				Muribaculum (33.2%)	*Muribaculum* sp. H5	2530393	
					*Muribaculum* sp. TLL-A4	2530390	
					*Muribaculum intestinale*	1796646	
Firmicutes (16.6%)	Bacilli (9.5%)	Lactobacillales (9.5%)	Enterococcaceae (0.3%)	Enterococcus (0.3%)	*Enterococcus faecalis*	1351	
			Lactobacillaceae (9.2%)	Lactobacillus (9.2%)	*Lactobacillus johnsonii*	33959	
					*Lactobacillus murinus*	1622	
					*Lactobacillus reuteri*	1598	
	Clostridia (7.1%)	Clostridiales (7.1%)	Clostridiaceae (0.3%)	Mordavella (0.3%)	*Mordavella* sp. Marseille-P3756	2086584	
			Lachnospiraceae (3.8%)	Lachnoclostridium (3.3%)	*[Clostridium] scindens*	29347	
					*Lachnoclostridium phocaeense*	1871021	
					*Lachnoclostridium* sp. YL32	1834196	
				Unknown (0.5%)	*Lachnospiraceae* bacterium GAM79	2109691	
			Oscillospiraceae (0.1%)	Unknown (0.1%)	*Oscillospiraceae* bacterium J115	2093857	
			Peptostreptococcaceae (1.6%)	Clostridioides (1.6%)	*Clostridioides difficile*	1496	
			Ruminococcaceae (0.9%)	Faecalibacterium (0.7%)	*Faecalibacterium prausnitzii*	853	
				Flavonifractor (0.2%)	*Flavonifractor plautii*	292800	
			Unknown (0.4%)	Unknown (0.4%)	*Clostridiales* bacterium CCNA10	2109688	
Proteobacteria (20.3%)	Epsilonproteobacteria (20.3%)	Campylobacterales (20.3%)	Helicobacteraceae (20.3%)	Helicobacter (20.3%)	Helicobacter typhlonius*	76936	
					Helicobacter hepaticus*	32025	
Actinobacteria (1.3%)	Actinobacteria (1.3%)	Bifidobacteriales (1.3%)	Bifidobacteriaceae (1.3%)	Bifidobacterium (1.3%)	*Bifidobacterium pseudolongum*	1694	
Verrucomicrobia (2.4%)	Verrucomicrobiae (2.4%)	Verrucomicrobiales (2.4%)	Akkermansiaceae (2.4%)	Akkermansia (2.4%)	*Akkermansia muciniphila*	239935	

^*^Pathogens

List of the twenty-four species with an average abundance higher than 1% ("Conventional-core species"), constituting 90.3% of bacteria present in the gut of non-SPF mice. Taxonomy from phylum to specie level is shown. The different lines represent the species included in the list. For each species there are columns that describe the taxonomy (phylum, class, order, family, genus, species, Species ID)

**Fig. 4:**
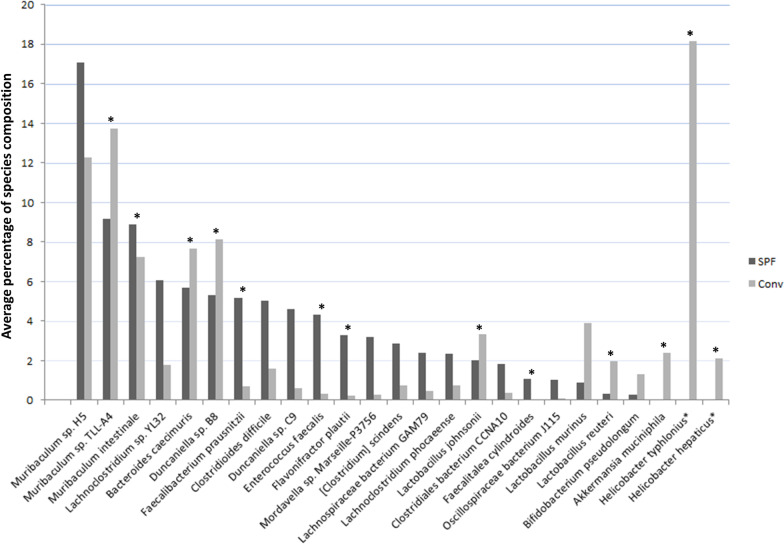
Comparison between abundant microorganisms in the SPF and non-SPF samples. Graph represents 18 species commonly shared between the two groups and 5 additional species (*Faecalitalea cylindroides*, *Bifidobacterium pseudolongum, Akkermansia muciniphila, Lactobacillus murinus,* and *Lactobacillus reuteri*) present at a very low fraction in one of the two groups. In addition, *Helicobacter typhlonius* and *Helicobacter hepaticus* constitute approximately 20% of the microorganisms in the non-SPF mice, while they are absent in the SPF mice. Asterisk (*) indicates species with a statistically significant differential abundance (*p* value < 0.05)

A comparison between the two lists of abundant microorganisms in the SPF and non-SPF samples showed that 18 species were commonly shared, while 5 species were present only in one of the two groups, at low percentage (Fig. 4), with the exception of *H. typhlonius* and *H. hepaticus* which represented about 20% of the microorganisms in the non-SPF mice; significantly, these species, which are considered pathogenic, were absent in the SPF mice.

### Pathogen detection by health monitoring assays and metagenomic shotgun sequencing

**Fig. 5:**
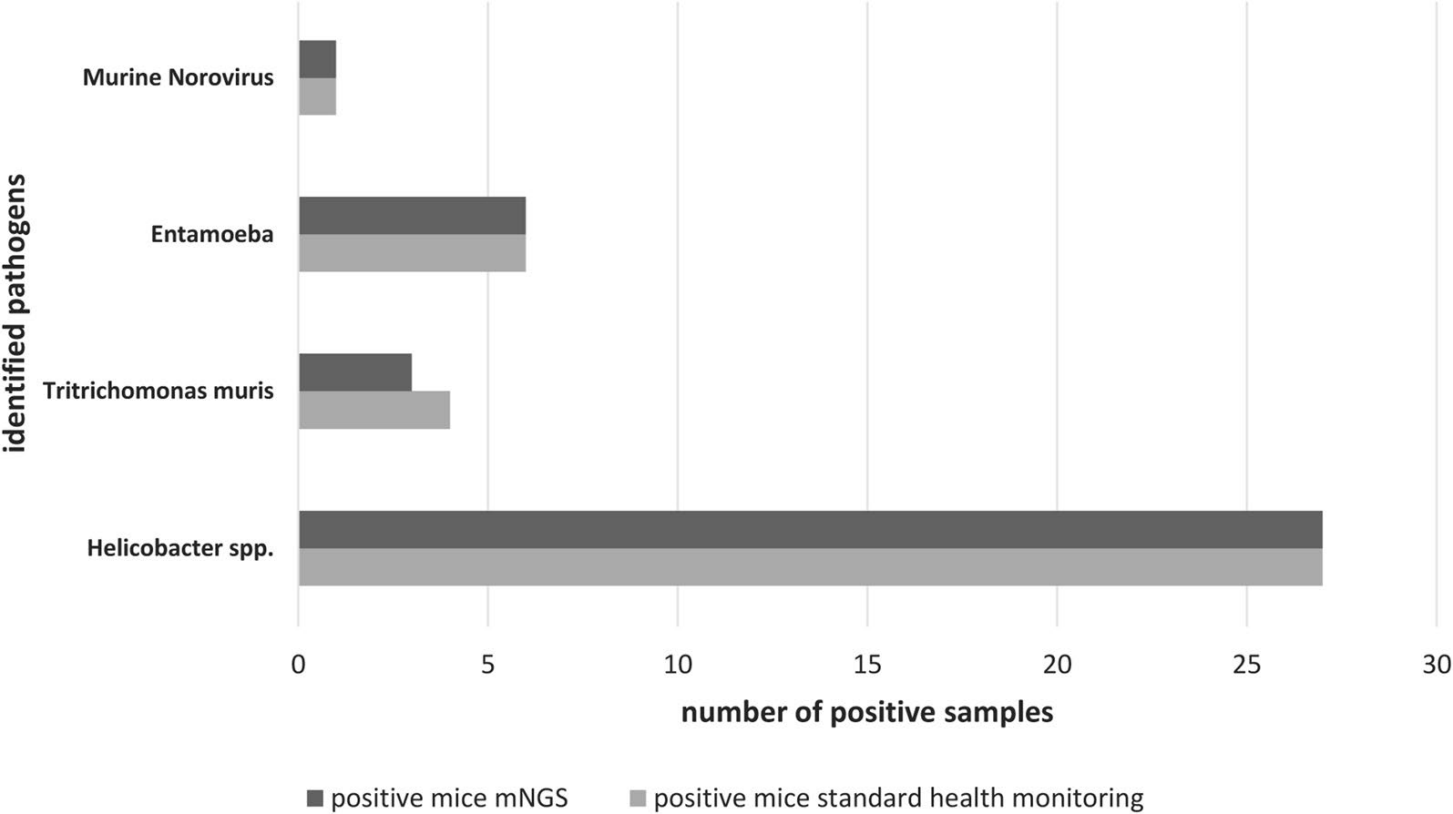
Comparison between metagenomic data and results from *health monitoring assays* employed for pathogens detection. Number of positive samples from standard health monitoring assays (light gray bars) and from mNGS analyses (dark gray bars) for the different pathogen species. x axis: number of samples positive for the indicated pathogen; y axis: identified pathogens. Results obtained by NGS are in line with data resulting from health monitoring assays as described in materials and methods except for one sample

**Fig. 6:**
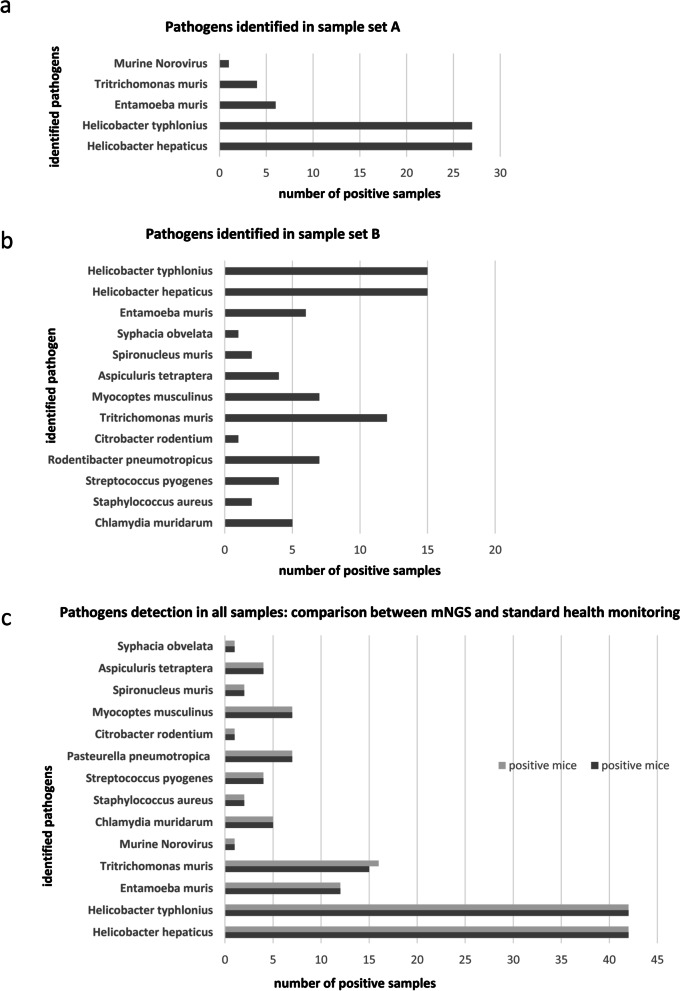
Summary of detected pathogens. Number of positive samples for different pathogen species detected by mNGS in **a** 27 non SPF fecal samples (sample set A) and in **b** additional 15 non SPF fecal samples (sample set B). **c** Comparison between metagenomic data (dark gray bars) and results from *health monitoring assays* (light gray bars) employed for pathogens detection in all 42 non-SPF mice. x axis: number of samples positive for the indicated pathogen. y axis: pathogen species. All data were confirmed by PCR and sequencing

SPF and non SPF sentinel mice (n = 21) were subjected to necropsy. Analyses were performed on different tissues as described in Materials and Methods and revealed the presence of pathogenic bacteria (Helicobacter species), of protozoa (Tritrichomonas muris, and Entamoeba muris) and of Norwalk virus, in non SPF animals. Conversely, no pathogens were identified in SPF animals. The results from standard monitoring assays were found to be in agreement with those obtained from the same animals with the mNGS approach. The additional 16 non SPF samples analyzed by mNGS and PCR revealed an overlapping of results: 16 were positive for *Helicobacter*, 1 for *Entamoeba muris* and 4 for both (Additional file 5) and (Fig. 5). Since the pathogenic species identified in these samples (hereinafter referred to as set A) were relatively few, to provide the proof-of-concept that mNGS is feasible for health monitoring, another set of 15 fecal samples (hereinafter referred to as set B) collected from animals of multiple non-SPF colonies were sequenced. (Additional file 6). In total, 14 different species of pathogens were identified in all non-SPF samples, belonging to pathogenic bacteria (*Helicobacter hepaticus, Helicobacter typhlonius, Chlamydia muridarum, Streptococcus pyogenes, Rodentibacter pneumotropicus, Citrobacter rodentium, Staphylococcus aureus*), intestinal protozoa (*Entamoeba muris*, *Tritrichomonas muris, Spironucleus muris*) nematoda (*Aspiculuris tetraptera, Syphacia obvelata*), eukaryotic parasites (*Myocoptes musculinus*) and RNA virus (*Norwalk virus*). (Fig. 6a, b). No discrepancy was found between mNGS results and those obtained by other techniques (as indicated in Materials and Methods) employed for standard monitoring assays, except for one sample positive for *Tritrichomonas muris* which resulted negative for mNGS. (Fig. 6c). No pathogens were identified in SPF animals, neither by mNGS, nor by the analyses carried out for health monitoring (Table 3). To verify the specificity and coverage of the two *Helicobacter* species, the reads of one non-SPF sample were directly mapped against *H. typhlonius* and *H. hepaticus* genomes. The *H. typhlonius* reads mapped over almost the whole genome (length 1.920.832 nt), with 1.594.236 nucleotides (83%) covered by at least one read, while The *H. hepaticus* reads were more dispersed along the genome (1.799.166 nt), with 500.064 (28%) nucleotides covered by at least one read (Additional file 8).

**Table 3:** List of pathogens identified in SPF and non-SPF mice

Superkingdom	Species ID	Species	Number positive mice (non-SPF set A)	Number positive mice (non-SPF set B)	Number positive mice (non-SPF total)	Number positive mice (SPF total)
Bacteria	32025	*Helicobacter hepaticus*	27	15	42	0
Bacteria	76936	*Helicobacter typhlonius*	27	15	42	0
Endo- and ectoparasites	545931	*Entamoeba muris*	6	6	12	0
Endo- and ectoparasites	5726	*Tritrichomonas muris*	4	12	16	0
Virus	28875	*Murine Norovirus*	1	0	1	0
Bacteria	83560	*Chlamydia muridarum*	0	5	5	0
Bacteria	1280	*Staphylococcus aureus*	0	2	2	0
Bacteria	1314	*Streptococcus pyogenes*	0	4	4	0
Bacteria	758	*Pasteurella pneumotropica*	0	7	7	0
Bacteria	67825	*Citrobacter rodentium*	0	1	1	0
endo- and ectoparasites	1046713	*Myocoptes musculinus*	0	7	7	0
endo- and ectoparasites	39710	*Spironucleus muris*	0	2	2	0
pinworm nematode	451377	*Aspiculuris tetraptera*	0	4	4	0
pinworm nematode	412127	*Syphacia obvelata*	0	1	1	0

List of the pathogenic species identified by mNGS and number of positive mice for each species. Each different line represents one pathogen species identified in the course of the study. For each species there are columns that describe a short taxonomy (superkingdom, species ID, species); the last four columns indicate the number of non-SPF mice of set A (non-SPF set A), non SPF-mice of set B (non-SPF set B) non-SPF mice all sets (non-SPF total), which were found positive for at least one pathogen. No pathogens were detected in SPF mice (SPF total). All data were confirmed by PCR and sequencing

**Fig. 7:**
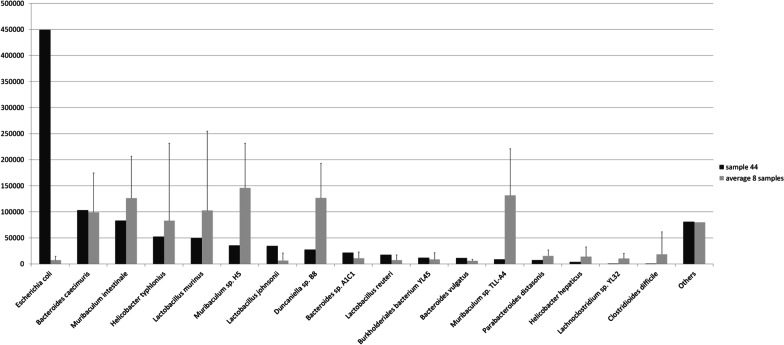
Comparison between *Escherichia coli* (*E. coli*) positive samples. Graph shows species detected in sample 44 (normalized counts) and in the remaining 8 non-SPF samples positive for *E. Coli* (normalized average). Species with values ​​greater than 100 ppm in at least one of the two groups of samples are represented. *E. Coli* represents 45% of the total reads in sample 44, while the average in the other samples is 0.7%. Among the most abundant species belonging to *Muribaculum* genus, all are reduced in sample 44, as a whole, from 40 to 13%

In addition, in a non-SPF sample (sample 44) we observed a high presence (45% of the total sample reads) of *Escherichia coli* (strain M8). Other eight samples were positive for *Escherichia coli,* but with a percent of the total sample reads ranging from 0.02 to 1.9%. In those eight samples, the most abundant species belonged to the Muribaculum genus (40.4% of the total reads, on average), while in sample 44 species of the Muribaculum genus were reduced to a 12.7% (Fig. 7).

Thus, results from this sample prove that microbiome analysis based on mNGS could produce a quantitative assessment of the relative abundance of each microbial species in the investigated samples able to reveal altered gut microbiome composition, which is not generally discovered by standard pathogen testing, qualitative and limited to the organisms recommended by FELASA.

## Discussion

A shotgun metagenomics NGS (mNGS) approach was performed to investigate the DNA and RNA microbiome from mice belonging to SPF or non-SPF conventional housing facilities. The goal was to define the gut microbiota composition as well as to uncover the presence of pathogens directly from fecal samples. In fact, mNGS technology allows sequencing of all nucleic acids derived from bacteria, viruses, fungi, and parasites that are present in the samples. Thus, in a single test, mNGS provides diagnostic information on pathogens, detailed description of the sample’s commensal microbiota, and provides a quantitative assessment of the relative abundance of each microbial species in the investigated samples via the sequencing read counts [28]. Metagenomic next-generation sequencing (mNGS) has recently been used to identify pathogenic infectious agents in human samples [25, 53] and its applicability to clinical practice for the diagnosis of human infections is clearly emerging [54, 55]. Validation of an mNGS test in the clinical setting requires to verify its accuracy through the comparison of the results with a gold standard technique, for example quantitative PCR; precision must be estimated by testing repeatability and reproducibility, by introducing sources of variations (separate batches, test on different days, different operators); robustness could be evaluated by analyzing the same sample in different experimental conditions, such as different amount of nucleic acids for library preparation. Presently, mNGS can be biased by two limitations: (1) the absence or the incomplete genome sequencing of all known microbes, which may impose a limit to their detection; (2) the fact that analytical algorithms for reads attribution and counting generally do not normalize for the size of each individual genome present in the database that is employed as reference. If not normalized, organisms with larger genomes can potentially produce a larger number of reads in the output and it is important to be aware of this issue when reporting species abundances [56]. Several other challenges still exists to routine development of metagenomic sequencing in the clinical setting, such as standardized clinical laboratory protocols, universally accepted reference standards, privacy concern, time frame required for clinical intervention and regulatory approval [57, 58]. However, addressing accuracy, precision, bias, and robustness, analytical and clinical validation of mNGS is indeed feasible and may offer distinct advantages in invasive procedure avoidance, cost effectiveness, and clinical outcomes [53].

The sequencing analysis of DNA and the reverse transcribed RNA from mouse fecal pellets was expected to detect genome fragments from bacteria, fungi, DNA and RNA viruses, and eukaryotic parasites in a single test. The present study demonstrates that this objective is indeed achievable.

In fact, the presence of pathogenic bacteria (*Helicobacter* [59]* hepaticus, Helicobacter typhlonius, Chlamydia muridarum, Streptococcus pyogenes, Rodentibacter pneumotropicus *[60]*, Citrobacter rodentium, Staphylococcus aureus*), intestinal protozoa (*Entamoeba muris*, *Tritrichomonas muris, Spironucleus muris*) nematoda (*Aspiculuris tetraptera, Syphacia obvelata*), eukaryotic parasites (*Myocoptes musculinus*) and RNA virus (*Norwalk virus*) has been demonstrated in some non-SPF samples*.*

Following shotgun NGS, the large quantity of genomes contributing to the microbiome could be identified by matching sequencing results with public databases through the use of available algorithms; the presence of pathogens, whenever present, could also be revealed. The robustness of this approach was high, as shown by sequencing five samples in duplicate. Sequencing was performed at different times and by different operators, obtaining an outcome almost identical in the two set of samples.

From a methodological point of view, the present study supports the concept that the shotgun metagenomic approach is more robust than the 16S amplicon sequencing. In fact, non-bacterial elements important for the health of the host, such as viruses, nematode and protozoa, are not detectable with 16S rRNA approach, while Shotgun sequencing, which is not based on the amplification of specific loci, supplies information on the total DNA content of microorganisms, including viruses, nematode and protozoa [61]. Moreover, since mNGS generates reads from all the parts of the microbial genomes and not only 16S, it also allowed a deeper characterization of the microbiome complexity, enabling the identification of microorganisms at the level of species or potentially even strain, which cannot be accomplished with the 16S analysis [61–63].

Furthermore, the mNGS approach has advantages compared to the classic microbiological methods. For example, it allows the identification of uncultivable or difficult to cultivate bacteria which could not be easily detected by classical bacteriology. Moreover, the use of mNGS overcomes the need for selective culture media and growth in anaerobic conditions.

Gut microbiota (GM) is important in maintaining the host health and its alterations may lead to disease [36, 64]. By being part of a “host-microbiome supra-organism” [24], the GM not only plays a protective role against pathogenic infections, but it also globally impacts the host health [65]. Currently, a number of studies describing the composition of the murine microbiota have been reported [47, 48, 66, 67]. These studies had distinct aims, employed different methods and various reference databases, and sometimes generated different results regarding the intestinal microbial composition. These differences can also be attributed to the different mouse facilities, strains, diet, and different exposure to environmental pathogens. We compared the results of other studies with ours (Additional file 9): in our experimental setting, the observed bacterial species mostly belonged to the phyla *Firmicutes* (45%), *Bacteroidetes* (53%), and, to a lesser extent to *Verrucomicrobia*, *Proteobacteria,* and *Actinobacteria*. Our data are in general agreement with the literature, where it is reported that the phyla *Firmicutes* and *Bacteroidetes* constitute up to 97% of the intestinal microbiota [12, 29, 66–70].

Among families, taxonomic analysis revealed as particularly abundant the family of *Muribaculaceae*, constituting more than 40% of the intestinal microbiota (42% in the non-SPF and 46% in the SPF group), confirming thus their status as dominant gut bacteria in mice [71, 72]. These data are apparently conflicting with some publications [48, 66, 70, 73] that report other families (*family S24-7 or Porphyromonadaceae*) but this discrepancy is mainly due to the sequencing system (16S versus shot gun metagenomics) and to the databases utilized for data analysis (SILVA [74] or RDP [75]) since family S24-7 or Porphyromonadaceae represent different denominations of taxa Muribaculaceae, whose name was proposed in 2019 [72].

We also compared the fecal microbiota of animals from conventional non-SPF housing facilities versus the composition observed in the SPF mice. The average percentage of *Firmicutes* in non-SPF mice is reduced to 19%, while that of *Proteobacteria* is increased to 21.3% for the abundance of *Helicobacter* species, which belong to *Proteobacteria*, generally comprising a small percentage (0.1%) of the SPF microbiota. The increase of *Proteobacteria* in the non-SPF animals compared to the SPF ones, is also reported in a recent study that analyzes the microbiota in animals with different degrees of exposure to environmental pathogens [48]. Therefore, the presence of pathogenic species not only constitutes a potential damage per se, but alters the composition of the microbiota.

Even though the definition of a “standard” mouse GM was not the goal of the present study, in our experimental setting we have observed that the largest part of SPF and non-SPF GM was made by a relatively small number of bacterial species, probably reflecting a “core” functional role of those species, independent of the housing conditions of the mice. This observation is in line with the decoupling between taxon and function and with the highly preserved functional capacity which are known in different types of microbial systems, host-associated or free-living [76–78]

In addition to the composition of the microbiota, we used the mNGS data to look for the presence of pathogenic microorganisms. We compared results from metagenomics analyses with those obtained by standard mouse health monitoring performed either using bacterial culture, PCR, serology, or microscopic observation of parasites. In most of the cases, mNGS analysis confirmed the presence of genomes belonging to the pathogenic organisms identified by the above-mentioned analyses, that is pathogenic bacteria (*Helicobacter hepaticus, Helicobacter typhlonius, Chlamydia muridarum, Streptococcus pyogenes, Rodentibacter pneumotropicus, Citrobacter rodentium, Staphylococcus aureus*), intestinal protozoa (*Entamoeba muris*, *Tritrichomonas muris, Spironucleus muris*) nematoda (*Aspiculuris tetraptera, Syphacia obvelata*), eukaryotic parasites (*Myocoptes musculinus*) and RNA virus (*Norwalk virus*).

Regarding pathogen surveillance, a number of aspects deserves further attention. First, the method allows the identification of bacterial taxa to the species level and may allow identification of specific genes, such as virulence factors and antibiotic resistance genes. For example, using the mNGS it was possible to distinguish between *Helicobacter* [59] *hepaticus* and *typhlonius*, which could not be achieved with the microbiological analysis routinely employed in health surveillance programs or with the 16S amplicon analysis [61–63]. Second, shotgun approach allowed the identification of the *Norwalk virus*, an RNA virus with high prevalence worldwide and commonly detected in laboratory mice [8, 79–83]. The detection of the genome confirms the presence of the virus, which cannot be guaranteed when employing only serological analyses; thus, the shotgun approach provides a higher level of certainty regarding the presence of viral pathogens. Third, this approach could also detect genome sequences from intestinal protozoa, like *Entamoeba muris*, *Tritrichomonas muris* and *Spironucleus muris* [84]. Infections with these protozoa are asymptomatic in immune-competent animals. While *Entamoeba, Spironucleus* and *Tritrichomonas* species were considered nonpathogenic members of the murine microbiome in laboratory mice, some studies suggested a role, at least for *Tritrichomonas muris*, in altering the immune response in disease models [4], potentially affecting results in research investigations. Moreover, the search for protozoa by means of molecular analysis is less operator-dependent than the search based on microscopic examination. Fourth, mNGS allowed the detection of parasite pinworms like *Aspiculuris tetraptera* and *Syphacia obvelata* that have a profound impact on health and research [85]. Fifth, a pathogenic fur mites as *Myocoptes musculinus* was identified, showing that this approach allows detection of also non-enteric microorganisms. We confirmed that is possible to detect fur mites from fecal pellets with a molecular approach, since mites and eggs can be ingested during grooming [86]. Other organisms as *Chlamydia muridarum* [87]*, Streptococcus pyogenes and Staphylococcus aureus* [88] which are not enteric, can possibly be found in the feces for the same reason. On the contrary, *Citrobacter rodentium* is an enteric bacterial pathogen which colonize the mouse intestinal mucosa [89] and *Pasteurella pneumotropica*, reclassified into the new genus *Rodentibacter* and renamed *Rodentibacter pneumotropicus* [60], colonizes the upper respiratory tract, the genital mucosa and the lower intestinal tract*.* Most of these microorganisms are present in the FELASA recommendations list since they are able to cause pathological signs especially in immunocompromised animals, thus providing a proof-of-concept that mNGS is a feasible approach for health monitoring.

This approach is able to detect any microorganism present mainly but not only in the gut of mice, as long as its nucleic acid sequence is presently available in public databases, avoiding thus the need to carry out multiple tests for the detection of each individual pathogen. It should be noted that if the goal of the test is to search specifically only for the *Norwalk virus, Rodentibacter*, *Syphacia* or any other specific microorganism, then RT-PCR and PCR are the methods of choice. However, in the case of mNGS a single test allows to analyze all pathogens, including the three mentioned above, with a lower risk of environmental contamination and to obtain numerous additional information, as previously described.

Lastly, the quantitative function of the NGS approach could uncover alterations in the composition of gut microbiota, as a result of the abnormal colonization by non-pathogenic organisms. For example, sequencing analysis of one mouse revealed that *E. coli* represented 45% of the total reads, thus it constituted the most abundant species in that animal. To investigate whether *E. coli,* colonizing that sample at such high levels, belonged to a pathogenic or a particularly virulent strain, the strain present in the sample was checked. The analysis revealed strain M8, originally identified from mice [90], which is non-pathogenic and is also present in other samples found positive for E.coli. Then, despite *E. coli* not being present in the FELASA list of pathogens, NGS analysis allows to distinguish non-pathogenic versus pathogenic *E. coli* strains, whose genomes differ by the presence of toxin genes. In this particular mouse, we could reveal gut colonization by a non-pathogenic *E. coli* strain, indicating a microbial imbalance, which could not be pinpointed by the traditional qualitative microbiological analyses.

In summary, this study demonstrates that the mNGS analysis can be utilized for the microbiome and pathogen monitoring of animals used for scientific research. In fact, the parallel sequencing of samples from several animals allows the identification of the microbiota on a taxonomic basis, up to the species level, providing more extensive and complete data compared to the monitoring of only a small number of microorganisms, which also depends on the use of sentinel animals.For instance, the method reveals bacteria such as Akkermansia, Faecalibacterium and Bifidobacterium, which might be beneficial for certain projects and models [92–95]. This approach could constitute a response to the general need of right tools to characterize the health status of animals housed in facilities with different microbiological status, in a broader sense beyond pathogen screening [96].

This study proposes mNGS as a tool for microbiome characterization and pathogen identification in laboratory animals, paving the way for its use in the clinical veterinary diagnostic practice and eventually in the epidemiology surveillance of pathogens that have caused recent zoonotic outbreaks of bacterial and viral origin [97–100]. As recently suggested, mNGS-based testing may in fact play a role in monitoring and tracking infectious disease outbreaks at the early stage [57]. The mNGS approach not only allows to highlight any infectious agent, including viruses, whose genome is present in public databases but it would also enable to highlight new pathogens originating from mutational or recombination events, provided their genome is known. It is worth to mention that only about 8% of the non-mouse sequence reads could align to microbial genomes, suggesting that they are absent in public databases because largely not yet sequenced.

In conclusion, considering the need to protect animal well-being and improve the reproducibility of biomedical preclinical studies, it appears reasonable to broaden the current concept of health monitoring of laboratory mice from “pathogen surveillance” to “microbiota surveillance”. This work provides the proof-of-concept that the use of a shotgun NGS metagenomics assay is a feasible and dependable approach.

## Materials and methods

### Mice and housing conditions

SPF and non-SPF C57BL/6NTacCnrm (B6N) mice, between 8 and 12 weeks of age, were used from facilities accredited by the Italian Ministry of Health in accordance with the Italian legislation Dlgs. 26/2014 and European directive 63/2010. All mice were bred in the Consiglio Nazionale delle Ricerche-European Mouse Mutant Archive (CNR-EMMA)-Infrafrontier (Monterotondo Scalo, Rome, Italy) in accordance with guidelines approved by the Institutional Animal Welfare Body (AWB) of CNR-IBBC/EMMA/Infrafrontier regarding animal breeding and in compliance with the European and Italian legislation. Mice were handled under BSL2 conditions in separate rooms, dedicated to SPF or non-SPF mice. Mice were housed in individually ventilated cages (Tecniplast, Gazzada, Italy) under a 12:12 light: dark cycle in microisolator cages under static conditions with autoclaved rodent chow (4RFN and EMMA 23, Mucedola, Settimo Milanese, Milano, Italy) and autoclaved tap water ad libitum and bedding (Scobis one, Mucedola, Settimo Milanese, Milano, Italy).

### Health monitoring assays

SPF and non SPF mice were routinely monitored to assess the health status of each microbiological unit according to the FELASA recommendations [83] every 3 months. Pathogens routinely monitored are listed in Additional file 5. Sentinel animals were maintained in a cage and received dirty bedding from the other cages of the colony, weekly, at every cage change. Sentinels represent the health status of the colony. All animals whose feces were subjected to NGS analysis, were also analyzed by the methods listed below.

Three to five sentinels from each rack or isolator were tested quarterly. Animals were sacrificed and subjected to necropsy, then examined for the presence of ectoparasites by direct microscopical examination of the skin and for the presence of endoparasite by observation of the caecum content. Blood was collected and serum was tested by ELISA serological method for the detection of viruses. ELISA kits from Charles River (USA) and Biotech Trading Partners (Encinitas, CA 92024, USA) were used according to manufacturer’s protocol. Positivity were confirmed by molecular tests. Nucleic acids were extracted from fecal pellets or from mesenteric lymph nodes.

*Norwalk Virus* was reverse transcribed using random primers and detected using primers ATAATTGGCAATTCCATCTCA and ATCACGCGGAGACCAGGA. PCR cycling conditions used were 95 °C for 2 min, followed by 50 cycles of 95 °C for 30 s, 56 °C for 30 s, and 72 °C for 1 min and a final extension time of 10 min at 72 °C. Product size was 563 bp. *Mouse Hepatitis Virus* (MHV), after reverse transcription using random primers was detected using primers AAGGTAGACGGTGTTAGCGG and TTTAACCCGCGCTCGGTTTG. PCR cycling conditions used were 95 °C for 2 min, followed by 50 cycles of 95 °C for 30 s, 60 °C for 30 s, and 72 °C for 1 min and a final extension time of 10 min at 72 °C. Product size was 241 bp. *Mouse Rotavirus* (EDIM), after reverse transcription using random primers, was detected using primers TTCCACCAGGAATGAATTGGAC and GGTCCTCACTTTACCAGCATG. PCR cycling conditions used were 95 °C for 2 min, followed by 50 cycles of 95 °C for 30 s, 62 °C for 30 s, and 72 °C for 1 min and a final extension time of 10 min at 72 °C. Product size was 118 bp. *Theiler's encephalomyelitis virus* (GDVII), after reverse transcription using random primers, was detected using primers CCCTACGGACCTTCTTTGTG and GAGCGGTACGTCAGTCCAGT. PCR cycling conditions used were 95 °C for 2 min, followed by 50 cycles of 95 °C for 30 s, 60 °C for 30 s, and 72 °C for 1 min and a final extension time of 10 min at 72 °C. Product size was 100 bp. *Mouse parvoviruses* (MVM and MPV) were detected using parvovirus generic primers TCAGTTCTAAAAATGATAAG and CCATTCATGCTGGACAAAC. PCR cycling conditions used were 95 °C for 2 min, followed by 50 cycles of 95 °C for 30 s, 48 °C for 30 s, and 72 °C for 1 min and a final extension time of 10 min at 72 °C. Product size was 500 bp.

Culture techniques were used for bacterial detection. Samples were collected from the intestine to determine bacterial flora of the digestive system and allowed to grow in a rich liquid culture medium overnight at 37 °C. Bacteria were then plated on rich and selective agar plates, colonies isolated and identified by classical bacteriology, gram stain, morphology and biochemical tests. Identification of relevant bacteria according to the FELASA recommendations was carried out and reported on the Health Monitoring Report produced quarterly for each animal colony and experimental unit. PCR was routinely used to detect *Helicobacter* species otherwise difficult to cultivate. Specifically, for fecal samples, organisms of the genus Helicobacter were detected using *Helicobacter* genus specific primers as described in [101] and species determined by sequencing, restriction enzyme analysis or by species specific primer amplification as described [102]. *Tritricomonas muris* and *Entamoeba muris* were detected by PCR. Primer used for *Tritricomonas muris* detection were CGATTGTTTCACTACGTTGAG and CAAACTCGCAGAGCTGGAAT, and the PCR cycling conditions used were 95 °C for 2 min, followed by 50 cycles of 95 °C for 30 s, 58 °C for 30 s, and 72 °C for 1 min and a final extension time of 10 min at 72 °C. Primer used for *Entamoeba* detection were CAGAATATCATCAAAAACAGTC and GAGAACCCACCAATTTCATCC and the PCR cycling conditions used were 95 °C for 2 min, followed by 50 cycles of 95 °C for 30 s, 55 °C for 30 s, and 72 °C for 1 min and a final extension time of 10 min at 72 °C. Product size were 330 bp and 340 bp respectively.

Primer used for *Chlamydia muridarum* were AGAGCCTACTTCTGGATGGATA and TTACCCAAGAGGGATTACAAGC and the PCR cycling conditions used were 94 °C for 5 min, followed by 35 cycles of 94 °C for 30 s, 58 °C for 30 s, and 68 °C for 30 s and a final extension time of 5 min at 72 °C. Product size was 116 bp. Primer used for *Streptococcus pyogenes* were TGCCTATGCCAGTGATTACG and GTCCCAGACACCTTGTTGAA and the PCR cycling conditions used were 95 °C for 15 min, followed by 35 cycles of 95 °C for 30 s, 55 °C for 30 s, and 72 °C for 40 s and a final extension time of 5 min at 72 °C. Product size was 132 bp. Primer used for *Rodentibacter pneumotropicus* were AGTATCGCGCTCTTCATTAGAC and CAGTCGTTCGGTAGGCTATTT and the PCR cycling conditions used were 95 °C for 15 min, followed by 35 cycles of 95 °C for 30 s, 55 °C for 30 s, and 72 °C for 40 s and a final extension time of 5 min at 72 °C. Product size was 109 bp. Primer used for *Citrobacter rodentium* were TAGCACTCATCGGCAACTTT and TAAAGTTAACAGAGCAGACAGTGA and the PCR cycling conditions used were 95 °C for 15 min, followed by 35 cycles of 95 °C for 30 s, 55 °C for 30 s, and 72 °C for 40 s and a final extension time of 5 min at 72 °C. Product size was 120 bp. Primer used for *Staphylococcus aureus* were TACGTATAATCATATTCATTTCT and TACGAATGATTGTATTTAAAA and the PCR cycling conditions used were 94 °C for 5 min, followed by 35 cycles of 94 °C for 30 s, 46 °C for 30 s, and 68 °C for 30 s and a final extension time of 5 min at 72 °C. Product size was 133 bp. Primer used for *Spironucleus muris* were GCTTCTGCCGCATCATCTA and GCCGTCTCTCATGCTCAC and the PCR cycling conditions used were 95 °C for 15 min, followed by 35 cycles of 95 °C for 30 s, 55 °C for 30 s, and 72 °C for 40 s and a final extension time of 5 min at 72 °C. Product size was 102 bp. Primer used for *Syphacia obvelata* were GAAGGTGAGAGTGAGTTGGTTAG and AGGACGAACACCAACAGAAATA and the PCR cycling conditions used were 94 °C for 5 min, followed by 35 cycles of 94 °C for 30 s, 56 °C for 30 s, and 68 °C for 30 s and a final extension time of 5 min at 72 °C. Product size was 695 bp. Primer used for *Aspiculuris tetraptera* were TGAAACCGCTGAGAAGGAAG and GAATCGCCCAACCAAACATATC and the PCR cycling conditions used were 95 °C for 15 min, followed by 35 cycles of 95 °C for 30 s, 55 °C for 30 s, and 72 °C for 40 s and a final extension time of 5 min at 72 °C. Product size was 132 bp. Primer used for *Myocoptes musculinus* were TTGATGGGTACCCTCGATTAT and GAATGAATCACATCAACAGAAG and the PCR cycling conditions used were 94 °C for 5 min, followed by 35 cycles of 94 °C for 30 s, 55 °C for 30 s, and 68 °C for 30 s and a final extension time of 5 min at 72 °C. Product size was 100 bp.

### Purification of nucleic acids

Samples employed in the study were fecal pellets from cages housed in SPF or in conventional non-SPF facilities. Fecal pellets were collected, transferred into a sterile, DNA-free Eppendorf tube, and were frozen at − 20 °C until use. Lysis Buffer (MC501C, Promega) was added to the fecal pellet, then transferred to a Lysing matrix B tube (MP Biomedicals), and homogenized following the manufacturer’s instructions in a Fast Prep FP120 (MP Biomedicals). Microbial nucleic acids (DNA and RNA) were isolated using the Promega Maxwell® RSC system (Promega) following the manufacturer’s instructions and frozen at − 20 °C. A negative control of sampling and extraction, consisted of an empty microisolator cage with the same rodent chow and bedding but no mice present in the cage. After a period of 4 weeks, a sample of chow and bedding was pulverized, transferred into a sterile, DNA-free Eppendorf tube, and frozen at − 20° C. The sample was subjected to the same DNA extraction procedure performed for the fecal samples and used for library preparation and sequencing as described below.

### Library preparation and sequencing

Nucleic acids were retro-transcribed to convert RNA to cDNA before library preparation. RNA was retro-transcribed using the following reagents: RevertAid H Minus Reverse Transcriptase (200 U/µL) (EP0451, Thermo Scientific); RNaseOUT™ Recombinant Ribonuclease Inhibitor (10777019, Invitrogen); Random Primers (48190011, Invitrogen); DTT 0.1 mM (P/N y00147, Thermo Scientific), 10 mM dNTP Mix (P/N y02256, Invitrogen). After incubation of RNA with Random Primers for 5 min at 70 °C, the other reagents were added and cDNA was synthetized at 37 °C for 1 h, followed by a 5 min-incubation at 94 °C.

Libraries were prepared using NEBNext Fast DNA Fragmentation & Library Prep Set for Ion Torrent (New England Biolabs # E6285L). Briefly, 50 ng of DNA were fragmented and end-repaired. Ion Torrent specific-motifs from Ion Xpress Barcode adapters (Thermo Fisher # 4,471,250) were ligated to both ends of DNA fragments. A size-selection, performed with Agencourt AMPure XP magnetic beads (Beckman Coulter #A63881), allowed to select 200 bp DNA fragments, that were successively amplified (9 cycles). Finally, libraries were cleaned-up through Agencourt AMPure XP beads and quantified using the Bioanalyzer 2100 instrument, with Agilent High Sensitivity DNA kit (Agilent # 5067-4626). No primer-dimers or adapter contamination was detected by Bioanalyzer tracing. Twenty-five libraries were pooled together and subjected to template preparation and sequencing, in accordance with Ion 540™ Kit-OT2 protocol (Thermo Fisher # A27753). Sequencing was performed on an Ion 540 chip (Thermo Fisher #A27765), using the Ion GeneStudio S5 System (Thermo Fisher), which yielded 1,5 gigabases (Gb) of high-quality data with an average of 3 × 10^6^ reads per sample (range: 2,071,086–3,998,008 reads per sample). Since each sample had an average of 3 million reads, the sensitivity was 1 out of about 3 million reads or 3 × 10^−7^.

### Bioinformatics and statistical analysis

Scheme of the employed pipeline is shown in Additional file 2. Reads shorter than 100 nucleotides were filtered out from raw FASTQ files, using PRINSEQ-lite 0.20.4 [103]. Reads matching the mouse genome were removed using bowtie2 [104] and samtools 1.4 [100]. The remaining reads were used to perform taxonomy calling at genus and species levels, using Kraken 2 [50], Bracken [51], and a database consisting of all the complete and draft genome sequences in GenBank Release 232 of archaea, bacteria, fungi, protozoa, virus and invertebrate endo- and ecto-parasites of mice (*Acantocephala*, *Annelida*, Helminths and *Nematoda*). Kraken2 was run with default parameters but with confidence score set to 0.5 in order to increase the precision. Each classified sequence (read) was attributed to its last known taxon (LKT). Genus and species with zero counts in all the samples were removed. The R programming language (version 3.5.0) was used to assemble all metagenomic data in a single table. The abundance of each taxon was plotted using the heat_tree function of the R package “metacoder” v. 0.3.3 [105] excluding low-abundance taxa (taxa accounting less than 1% of reads in all the samples). Data were subjected to the D’Agostino–Pearson omnibus normality test. Analyses and data plot were performed with Prism version 6.0f (GraphPad Software) unless otherwise stated. To evaluate the statistical significance between SPF and non-SPF animals we applied the DESeq2 bioconductor package [106].

## Supplementary Information

Additional file 1. Experimental design: test approaches and expected outcomes. Samples were from mice housed in a SPF housing facility or in a non-SPF facility. Twenty one mice (10 SPF and 11 non SPF) were sentinel animals included in the institutional health monitoring program, routinely monitored to assess the health and microbiological status of the colony. Sixteen mice belonged to breeding colonies of a conventional (non-SPF) facility and thus only fecal pellets were analyzed. The figure shows the types of methods and the expected outcomes from control and test samples from each type of assay

Additional file 2. Overview of the bioinformatics pipeline. Reads from raw FASTQ files were filtered by length using PRINSEQ-lite; putative mouse reads were removed using bowtie2 and samtools 1.4. The remaining reads were used to perform taxonomy calling at genus and species levels, using Kraken 2 [50], Bracken [51], and a database consisting of all the complete and draft genome sequences in GenBank Release 232 of archaea, bacteria, fungi, protozoa, virus and invertebrate endo- and ecto-parasites of mice (*Acantocephala*, *Annelida*, Helminths and *Nematoda*).

Additional file 3. Raw data form NGS analyses. The different lines represent the different species identified with the normalized counts. For each species there are 8 columns that describe the taxonomy (superkingdom, phylum, class, order, family, genus, species, Species ID). The following columns identify the reads of each sample.

Additional file 4. Raw data form NGS analysis of the negative control. The different lines represent the different species identified with the normalized counts. For each species there are 8 columns that describe the taxonomy (superkingdom, phylum, class, order, family, genus, species, Species ID). The following columns identify the reads of the negative control sample and the average reads of the fecal samples.

Additional file 5. FELASA list of pathogens investigated in the course of the study: comparison between standard vs NGS analyses. Each different line represents the species included in the FELASA list of pathogens. For each species there are 6 columns that describe a short taxonomy (superkingdom, NCBI Taxon ID, species); the last two columns indicates the number of positive mice identified by standard or NGS analyses as described in Matherial and Methods.

Additional file 6. Raw data form NGS analyses (sample set B). The different lines represent the different species identified with the normalized counts. For each species there are 8 columns that describe the taxonomy (superkingdom, phylum, class, order, family, genus, species, Species ID). The following columns identify the reads of each sample.

Additional file 7. Differences between SPF and non-SPF mice at phylum and family level. Each line represents phylum (average greater than 100 ppm in at least one of the two groups of samples) or family (average above 100 ppm in both groups of samples, except for *Helicobacteraceae* which are absent in the SPF mice) identified in SPF and non-SPF samples. Pvalues have been calculated by DSEQ2 in Bioconductor. Families and phyla whose differences were statistically significant show a pvalue< 0,05.

Additional file 8. Reads alignment to H. typhlonius and H. hepaticus genomes. Image shows reads alignment to illustrative portions of the *Helicobacter* genome in one non-SPF sample: (a) reads (horizontal gray bars) mapped to a 2,308 bp region of *H. typhlonius* genome (nucleotides from nt 694,600 to 696,600 are indicated); (b) detail of a 145 bp region and reads mapped to that region. (c) reads (horizontal gray bars) mapped to a 2,162 bp region of the *H. hepaticus* genome (nucleotides from 488,600 to 490,400 are indicated); (d) detail of a 136 bp region and reads mapped to that region. The *H. typhlonius* reads mapped over almost the whole genome (length 1.920.832 nt), with 1.594.236 nucleotides (83%) covered by at least one read. The *H. hepaticus* reads were more dispersed along the genome (1.799.166 nt), with 500.064 (28%) nucleotides covered by at least one read.

Additional file 9. Taxonomic composition of gut microbiota at phylum level: comparison with other studies. Comparison between the present study and four published studies (indicated with author’s name and [ref.]). For each study are indicated: sequencing method; health/ microbiological status of mice; mice strain; type of sample (DNA source); relative abundance of each of the four most represented phyla of the gut microbiota.

## Data Availability

All data generated or analysed during this study are included in this published article and its supplementary information files. The dataset supporting the conclusion of this article is included within the article and its additional files.
