# Supplementary material for: A comprehensive approach for microbiota and health monitoring in mouse colonies using metagenomic shotgun sequencing
Source: Anim Microbiome. 2021 Jul 29;3:53. doi: 10.1186/s42523-021-00113-4 (PMC8323313; doi:10.1186/s42523-021-00113-4)
Supplement: Supplementary file 1 — Experimental design: test approaches and expected outcomes. Samples were from mice housed in a SPF housing facility or in a non-SPF facility. Twenty one mice (10 SPF and 11 non SPF) were sentinel animals included in the institutional health monitoring program, routinely monitored to assess the health and microbiological status of the colony. Sixteen mice belonged to breeding colonies of a conventional (non-SPF) facility and thus only fecal pellets were analyzed. The figure shows the types of methods and the expected outcomes from control and test samples from each type of assay [file 42523_2021_113_MOESM1_ESM.pdf]

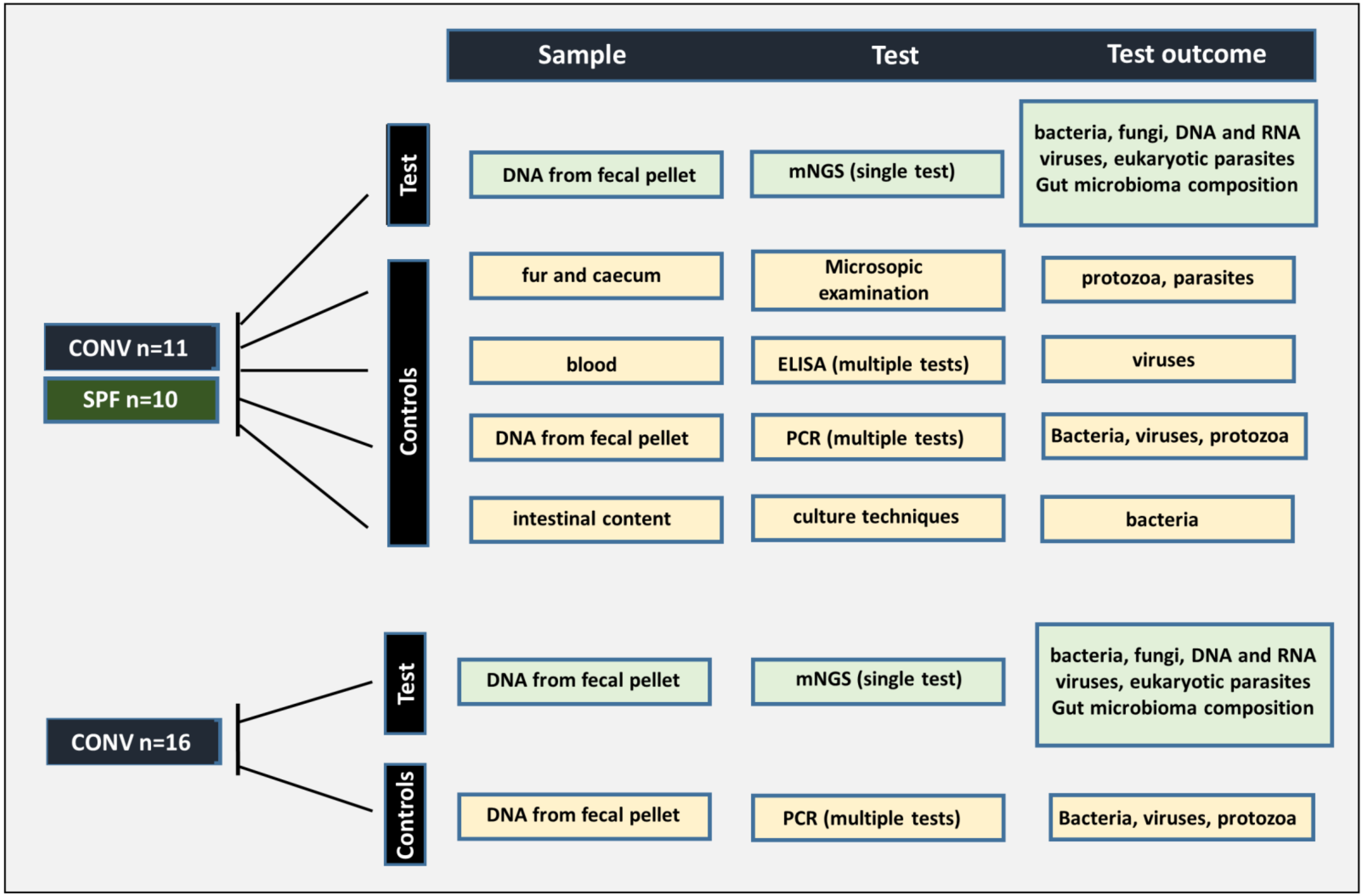

Figure S1. ***Experimental design: test approaches and expected outcomes.*** Samples were from mice housed in a SPF housing facility or in a non-SPF facility. Twenty one mice (10 SPF and 11 non SPF) were sentinel animals included in the institutional health monitoring program, routinely monitored to assess the health and microbiological status of the colony. Sixteen mice belonged to breeding colonies of a conventional (non-SPF) facility and thus only fecal pellets were analyzed. The figure shows the types of methods and the expected outcomes from control and test samples from each type of assay.
