# Supplementary material for: A comprehensive approach for microbiota and health monitoring in mouse colonies using metagenomic shotgun sequencing
Source: Anim Microbiome. 2021 Jul 29;3:53. doi: 10.1186/s42523-021-00113-4 (PMC8323313; doi:10.1186/s42523-021-00113-4)
Supplement: Supplementary file 2 — Overview of the bioinformatics pipeline. Reads from raw FASTQ files were filtered by length using PRINSEQ-lite; putative mouse reads were removed using bowtie2 and samtools 1.4. The remaining reads were used to perform taxonomy calling at genus and species levels, using Kraken 2 [50], Bracken [51], and a database consisting of all the complete and draft genome sequences in GenBank Release 232 of archaea, bacteria, fungi, protozoa, virus and invertebrate endo- and ecto-parasites of mice (Acantocephala, Annelida, Helminths and Nematoda). [file 42523_2021_113_MOESM2_ESM.pdf]

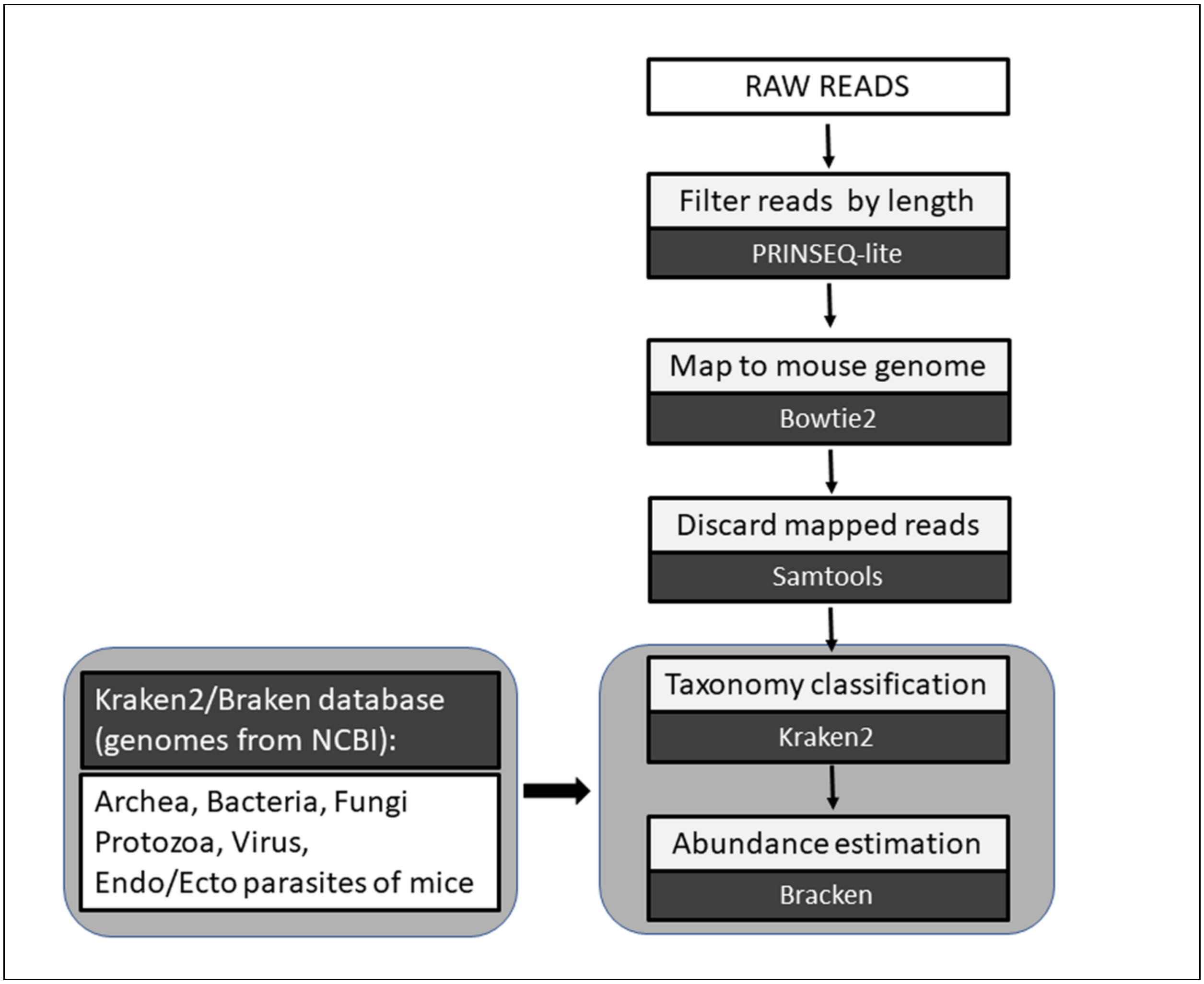

Supplementary Figure 1. Overview of the bioinformatics pipeline. Reads from raw FASTQ files were filtered by length using PRINSEQ-lite; putative mouse reads were removed using bowtie2 and samtools 1.4. The remaining reads were used to perform taxonomy calling at genus and species levels, using Kraken 2 (50), Bracken (93), and a database consisting of all the complete and draft genome sequences in GenBank Release 232 of archaea, bacteria, fungi, protozoa, virus and invertebrate endo- and ecto-parasites of mice (*Acantocephala*, *Annelida*, Helminths and *Nematoda*).
