# Supplementary figures and images for: A comprehensive approach for microbiota and health monitoring in mouse colonies using metagenomic shotgun sequencing
Source: Anim Microbiome. 2021 Jul 29;3:53. doi: 10.1186/s42523-021-00113-4 (PMC8323313; doi:10.1186/s42523-021-00113-4)

**Helicobacter typhlonius**

**a**

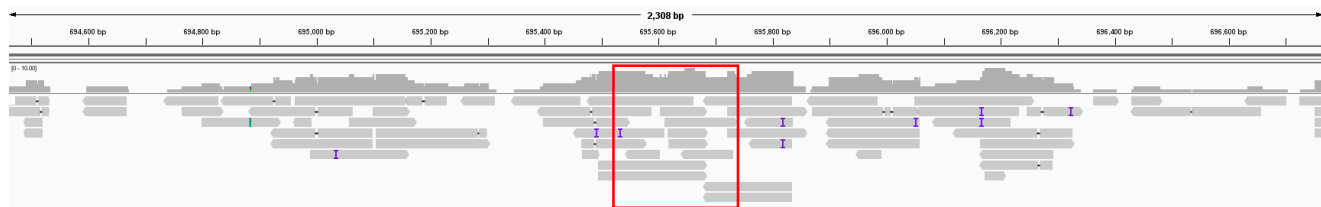

**b**

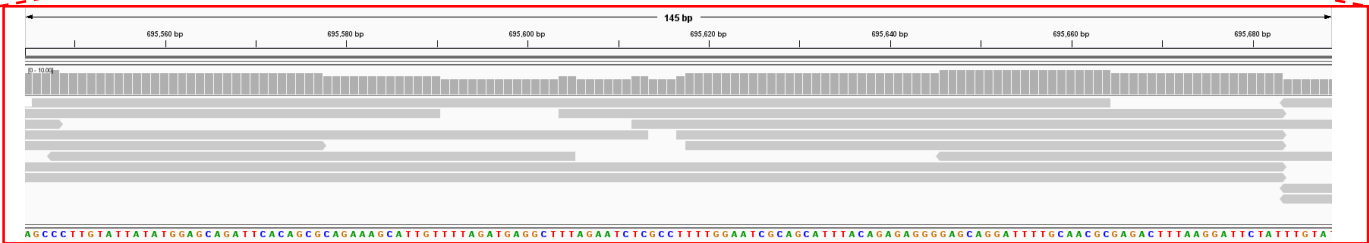

**Helicobacter hepaticus**

**c**

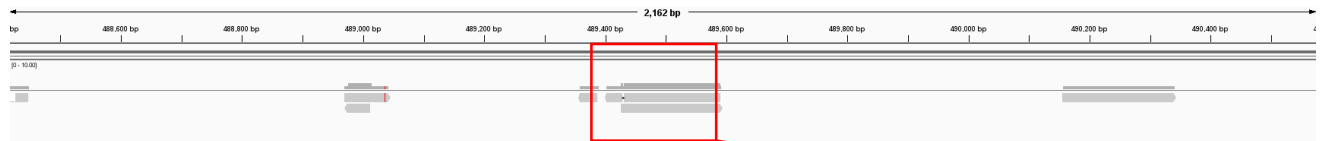

**d**

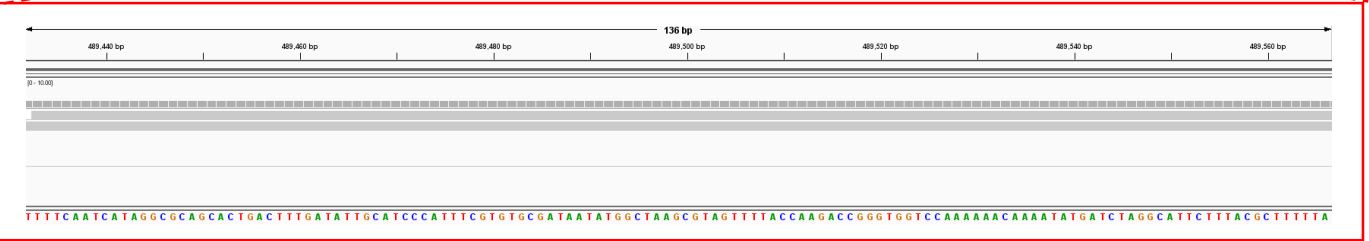

Supplement: Supplementary file 8 — Reads alignment to H. typhlonius and H. hepaticus genomes. Image shows reads alignment to illustrative portions of the Helicobacter genome in one non-SPF sample: (a) reads (horizontal gray bars) mapped to a 2,308 bp region of H. typhlonius genome (nucleotides from nt 694,600 to 696,600 are indicated); (b) detail of a 145 bp region and reads mapped to that region. (c) reads (horizontal gray bars) mapped to a 2,162 bp region of the H. hepaticus genome (nucleotides from 488,600 to 490,400 are indicated); (d) detail of a 136 bp region and reads mapped to that region. The H. typhlonius reads mapped over almost the whole genome (length 1.920.832 nt), with 1.594.236 nucleotides (83%) covered by at least one read. The H. hepaticus reads were more dispersed along the genome (1.799.166 nt), with 500.064 (28%) nucleotides covered by at least one read. [file 42523_2021_113_MOESM8_ESM.pdf]
